# Tako-tsubo-like left ventricular dysfunction in a patient with COVID-19 demonstrated by non-invasive multi-modality imaging

**DOI:** 10.1007/s12350-020-02367-y

**Published:** 2020-10-01

**Authors:** Kazuhiro Fujiyoshi, Junya Ako, Kohki Ishida, Miwa Ishida, Yoshiyasu Minami, Takayuki Inomata

**Affiliations:** 1 Department of Cardiovascular Medicine, Kitasato University Kitasato Institute Hospital, 5-9-1 Shirokane, Minato-ku, Tokyo, 108-8642 Japan; 2 Department of Cardiovascular Medicine, Kitasato University School of Medicine, Sagamihara, Kanagawa Japan

Tako-tsubo-like left ventricular (LV) dysfunction has been reported to occur concomitantly with COVID-19.^1^ The diagnosis of tako-tsubo can be a challenge when invasive catheterization cannot be readily indicated in patients with COVID 19.

**Figure 1:**
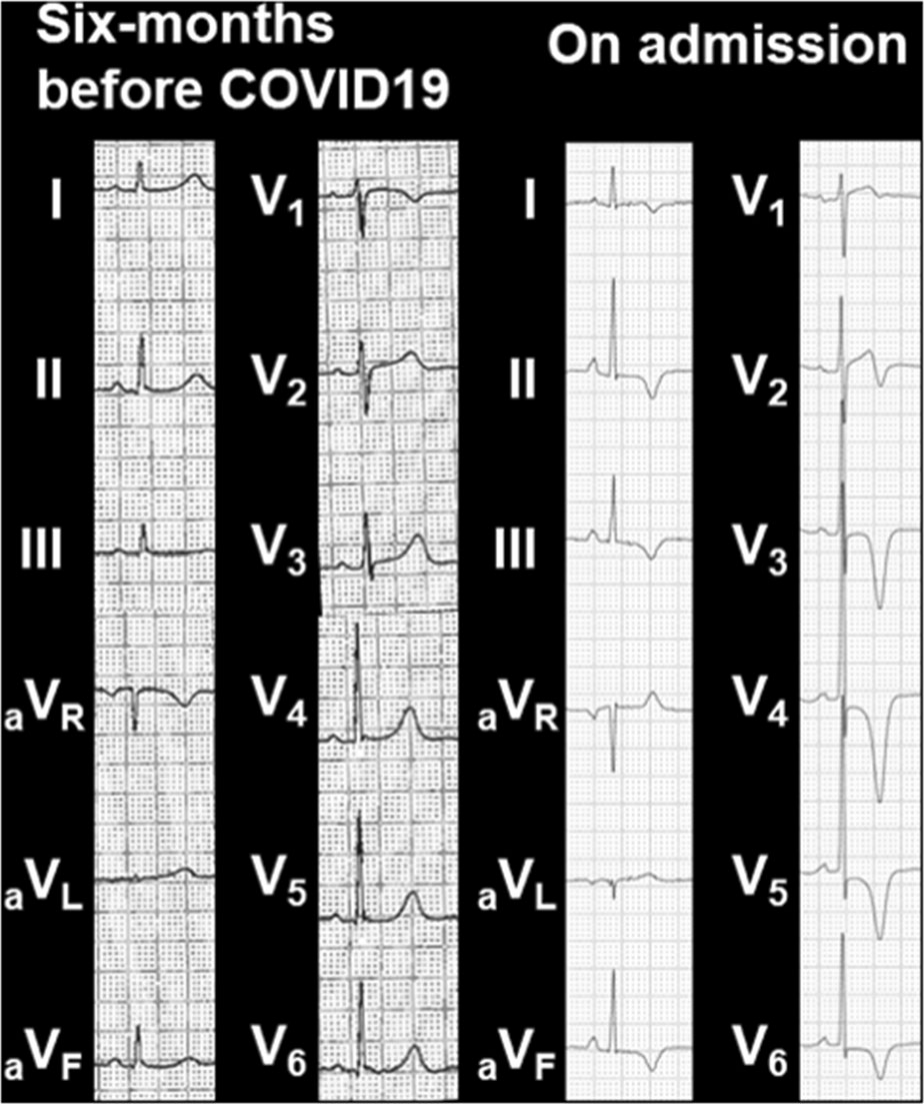
Electrocardiogram before 6 months of COVID-19 showing normal, and the electrocardiogram on admission revealing deep T-wave inversions in all precordial leads

**Figure 2:**
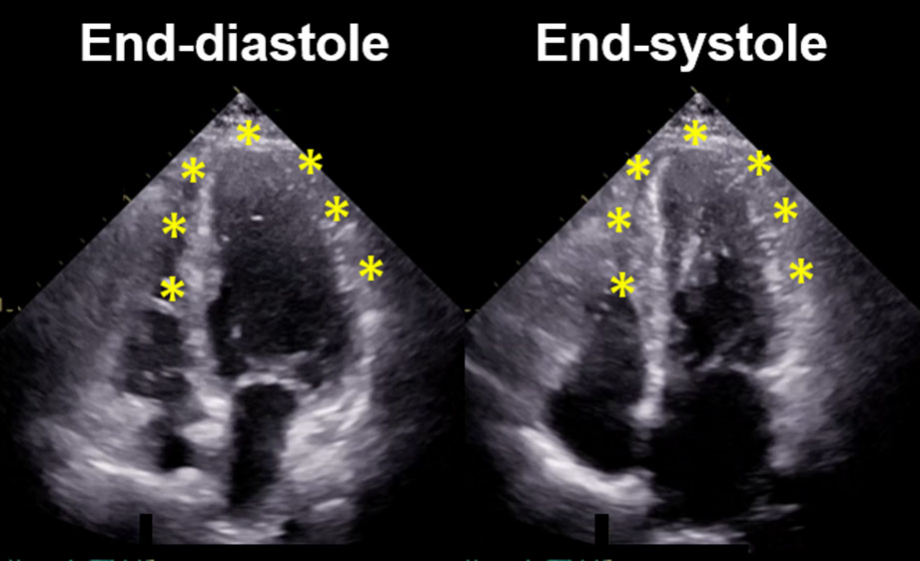
Apical four-chamber view of transthoracic echocardiographic images in diastole and systole on admission. Echocardiographic imaging demonstrating hypokinesis with hypertrophy in the apical region (asterisk) like an appearance of apical hypertrophic cardiomyopathy, and hyperkinesis in the basal region with estimated LV ejection fraction of 58%

**Figure 3:**
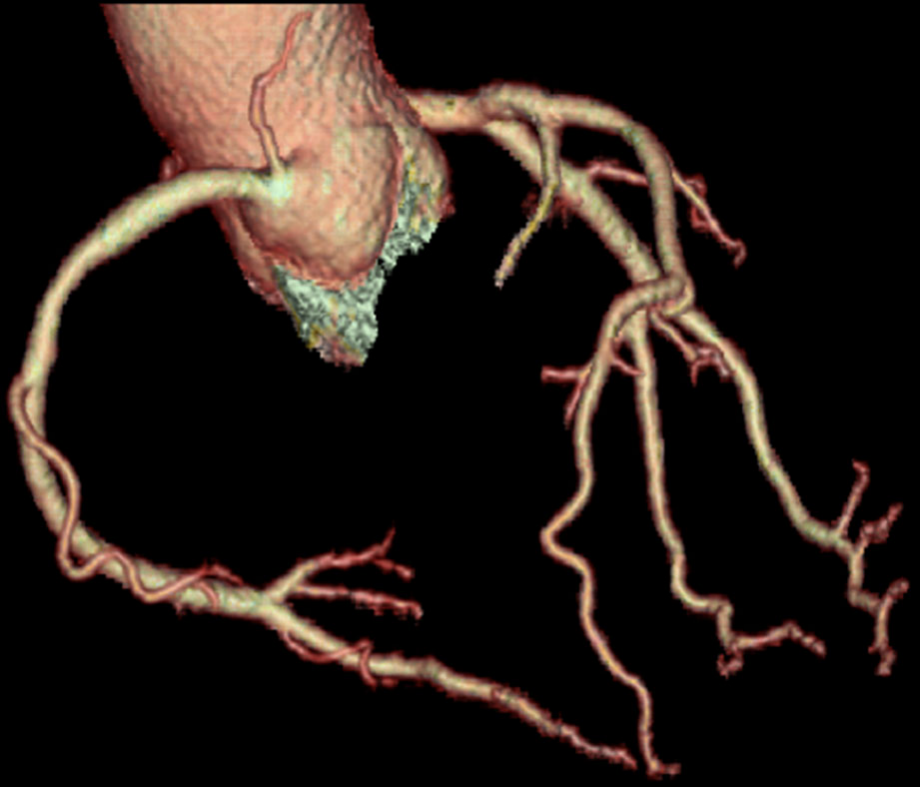
Computed tomography angiography image on admission showing no obstruction or stenosis in the epicardial coronary arteries despite of the electrocardiogram change showing Fig. 1

**Figure 4:**
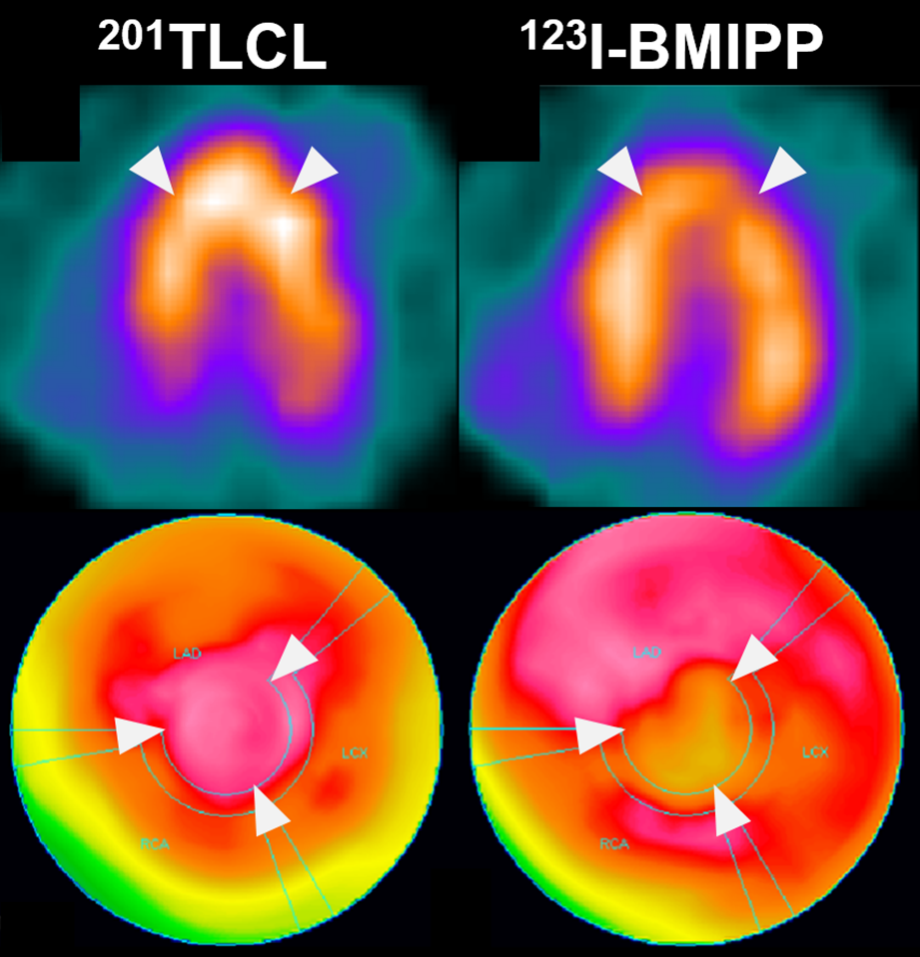
Dual isotope scintigraphy images at four days after admission showing increased ^201^TLCL uptake and decreased ^123^I-BMIPP uptake at LV apex (white arrow). This mismatch of nuclear uptake findings suggested tako-tsubo-like LV dysfunction. ^201^TLCL, thallium-201 chloride; ^123^I-BMIPP, iodine-123-beta-methyl-p-iodophenyl-pentadecanoic acid

**Figure 5:**
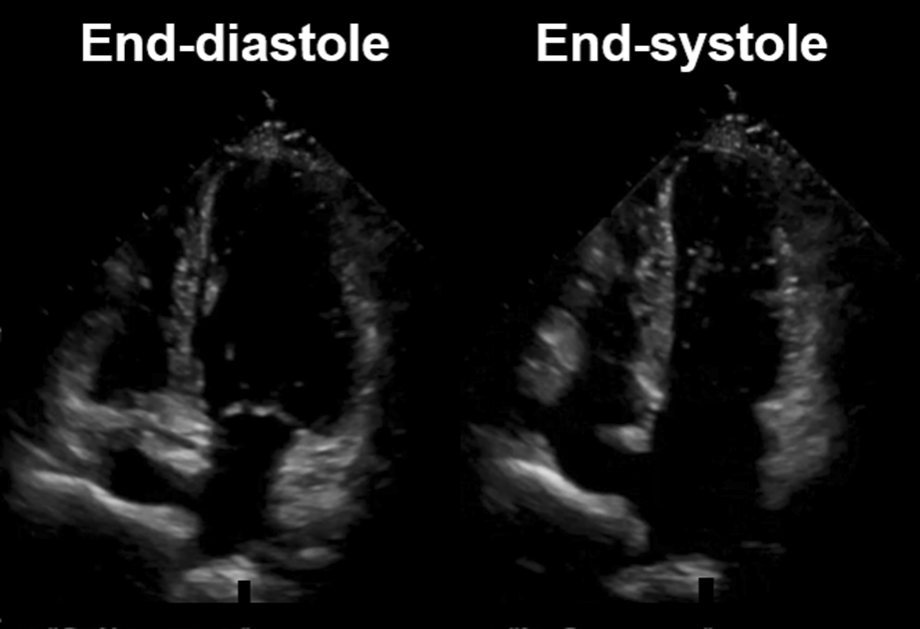
Apical four-chamber view of transthoracic echocardiographic images in diastole and systole 2 weeks after discharge. Echocardiographic imaging demonstrating normal LV wall motion and trivial apical hypertrophy with estimated LV ejection fraction of 63%

A 71-year-old woman with history of hypertension and anxiety disorder visited our hospital because of trivial fever and shortness of breath lasting for 2 months. Computed tomography revealed trivial peripheral consolidations and nasopharyngeal swab for SARS-CoV-2 revealed positive. Laboratory findings showed slightly elevated cardiac troponin (38.4 pg/mL). Electrocardiography on admission revealed deep T-wave inversions in all precordial leads (Figure 1). Echocardiography demonstrated hypokinesis with hypertrophy in the apical region (asterisk, Figure 2) and hyperkinesis in the basal region with estimated LV ejection fraction of 58%. Coronary computed tomography angiography was normal (Figure 3). Dual-isotope scintigraphy revealed increased thallium-201 chloride (^201^TLCL) uptake and decreased iodine-123-beta-methyl-p-iodophenyl-pentadecanoic acid (^123^I-BMIPP) uptake at LV apex (arrow, Figure 4). The patient was diagnosed with Tako-tsubo-like LV dysfunction based on those findings. Medical management was based on careful observation followed clinical improvement, and she was discharged on hospital day 12. Two weeks after discharge, electrocardiogram of T-wave inversions became shallow and echocardiographic findings improved to normal LV wall motion with trivial apical hypertrophy (Figure 5).

Herein, this is a case that nuclear medicine might be helpful to diagnose safely with tako-tsubo-like LV dysfunction in a patient with COVID-19 infection.^2^ Given the clinical presentation, electrocardiographic findings, biomarker profiles and left ventricular abnormal findings, the differential diagnosis included ischemic heart disease, apical hypertrophic cardiomyopathy and tako-tsubo-like LV dysfunction.^3^ These finding was compatible with tako-tsubo-like LV dysfunction in recovery phase. Since tako-tsubo-like LV dysfunction is hard to be differentiated from acute coronary syndrome, cautions should be exercised when choosing appropriate diagnostic measures.
